# Association between maternal blood lead levels and prevalence of dental caries in the primary dentition of children

**DOI:** 10.1265/ehpm.25-00188

**Published:** 2025-11-22

**Authors:** Yoshie Nagai-Yoshioka, Ryota Yamasaki, Reiko Suga, Mayumi Tsuji, Reiji Fukano, Kiyoshi Yoshino, Seiichi Morokuma, Wataru Ariyoshi, Masanori Iwasaki

**Affiliations:** 1 Department of Health Promotion, Division of Infections and Molecular Biology, Kyushu Dental University, Kitakyushu, Japan; 2 Regional Center for Japan Environment and Children’s Study (JECS), University of Occupational and Environmental Health, Kitakyushu, Japan; 3 Department of Environmental Health, School of Medicine, University of Occupational and Environmental Health, Kitakyushu, Japan; 4 Department of Pediatrics, School of Medicine, University of Occupational and Environmental Health, Kitakyushu, Japan; 5 Department of Obstetrics and Gynecology, School of Medicine, University of Occupational and Environmental Health, Kitakyushu, Japan; 6 Department of Health Sciences, Graduate School of Medical Sciences, Kyushu University, Fukuoka, Japan; 7 Department of Preventive Dentistry, Faculty of Dental Medicine and Graduate School of Dental Medicine, Hokkaido University, Sapporo, Japan

**Keywords:** Dental caries, Heavy metal, Lead, Oral health, Japan Environment and Children’s Study

## Abstract

**Background:**

Dental caries is a chronic childhood disease and one of the most prevalent public health problems worldwide. Lead is a heavy metal that is taken up by the teeth and bones. However, the association between lead exposure during pregnancy, when the tooth germs are formed, and the prevalence of dental caries in the primary dentition remains unclear. This study aimed to examine the association between maternal blood lead levels and the prevalence of dental caries in the primary dentition of children.

**Methods:**

This cross-sectional study was conducted as an Adjunct Study to the Japan Environment and Children’s Study (JECS), which is an ongoing nationwide birth-cohort study. Among children participating in the JECS at the University of Occupational and Environmental Health Sub-Regional Center, those aged 7–8 years underwent oral examination and questionnaire administration. The dft (i.e., sum of the number of decayed and filled primary teeth) was then determined. The dft numerically expresses the dental caries prevalence in the primary dentition (larger value indicates more prevalent dental caries). Poisson regression analyses with robust standard errors were performed to evaluate the association between maternal blood lead levels during pregnancy, measured using frozen samples, and the dft.

**Results:**

The study included 139 children, of whom 54.7% were girls, and 89.2% were 7 years old. The median maternal blood lead level was 6.1 ng/g (25–75 percentile, 5.0–7.3). The median dft was 0 (25–75 percentile, 0–4). After adjusting for covariates including age, sex, and oral health status and behavior, maternal blood lead levels were significantly associated with increased dft (prevalence ratio, 1.6; 95% confidence interval, 1.3–1.8; per one standard deviation increase in natural log-transformed maternal blood lead levels).

**Conclusions:**

This study found an association between maternal blood lead levels and the prevalence of dental caries in the primary dentition of children aged 7–8 years. Maternal exposure to lead during mid- to late-term pregnancy may affect the caries susceptibility of children after birth.

**Supplementary information:**

The online version contains supplementary material available at https://doi.org/10.1265/ehpm.25-00188.

## Graphical Abstract

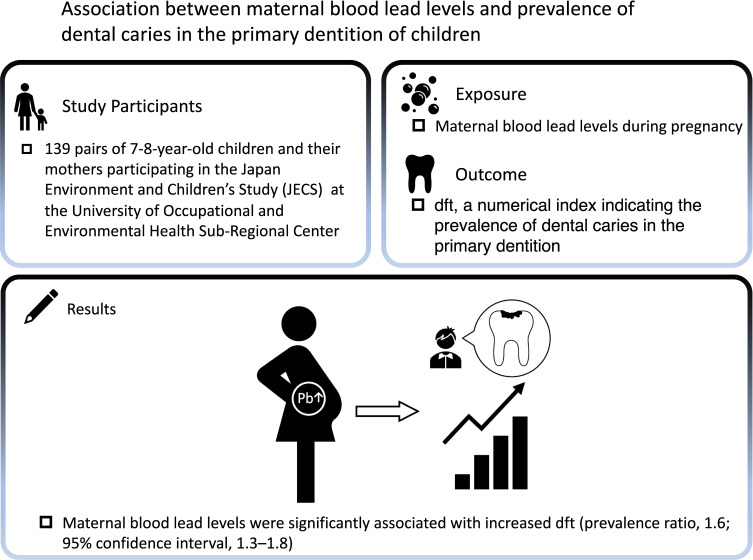

## Background

The primary teeth serve multiple purposes, including mastication, space maintenance, and speech development [1]. Dental caries is caused by complex interactions between acid-producing bacteria, carbohydrates, and multiple host factors, including the teeth and saliva [2]. Dental caries in the primary teeth is a serious chronic childhood disease and a highly prevalent public health problem worldwide, according to the World Health Organization [3]. Childhood dental caries is characterized by rapid progression, childhood distress, repeated antibiotic prescriptions, severe pain, sepsis, and sleep disturbances [1]. Moreover, decay in the primary teeth is a strong risk factor for dental caries in the permanent teeth [4, 5].

Heavy metals, including arsenic, cadmium, mercury, zinc, and lead, are natural metallic elements that are denser than water and are found throughout the Earth’s crust [6]. Heavy metals are widely distributed in the environment because of their diverse uses in industry, agriculture, and medicine. These heavy metals are highly toxic and have become an important public health priority owing to concerns about their effects on human health [7, 8]. For example, lead is associated with a lower intelligence quotient, especially when exposure occurs during childhood [9], and neurobehavioral disorders in children [10–12]. Furthermore, some heavy metals may pass through the placenta to the fetus and affect fetal growth [13]. Previous epidemiological studies have shown that lead exposure during pregnancy is inversely associated with birth weight, birth height, head circumference, and gestational week [14, 15].

In addition to causing systemic health problems, lead is taken up by the teeth and bones and affects tooth demineralization [16]. Although the detailed mechanism by which lead exposure affects dental caries remains unknown, *in vivo* studies have shown that calcium in hydroxyapatite is replaced by lead during tooth formation [17]. Epidemiological studies have reported a positive association between blood lead levels and the number of decayed teeth in schoolchildren [18]. Furthermore, positive associations have been reported between the salivary and enamel lead levels and caries severity [19]. However, to the best of our knowledge, no study has evaluated the association between lead exposure during pregnancy, when the primary teeth and permanent tooth germs are formed, and dental caries in the primary teeth of children.

Therefore, this study investigated the association between maternal blood lead levels and the prevalence of dental caries in the primary dentition of children aged 7–8 years. Specifically, we hypothesized that high maternal blood lead levels are associated with a larger number of decayed teeth in the primary dentition of children.

## Methods

### Study population

This study is an Adjunct Study to the Japan Environment and Children Study (JECS). The JECS is a government-funded, nationwide, prospective birth cohort study initiated in January 2011 to investigate the impact of environmental chemicals on child health. The JECS recruited pregnant women who lived in the study area between January 2011 and March 2014, and a total of 104,062 pregnant women from 15 study regions in Japan were enrolled. Participants’ biological samples, such as blood, were collected during the first trimester, second/third trimester, and at delivery. The JECS protocol and profiles have been described in detail elsewhere [20, 21].

This study was conducted from July 2019 to September 2019 at the University of Occupational and Environmental Health in Kitakyushu, Fukuoka, Japan, which participated in the JECS. In this study, survey instructions were mailed to 512 pairs of parents and children eligible for the 8-year follow-up of the JECS survey conducted in Kitakyushu City, Fukuoka Prefecture, Japan.

Of these, 159 participants who were examined during the follow-up survey were approached to participate in an Adjunct Study JECS survey conducted in Fukuoka. A total of 156 individuals participated in this additional survey. After excluding participants with missing data on oral health status, fluoridation experience, and maternal blood lead levels, data from 139 participants were included in the analysis.

### Measurement of maternal blood lead level

Maternal blood lead levels (ng/g) were measured using frozen whole-blood samples collected during the second/third trimester of pregnancy through inductively coupled plasma mass spectrometry. Detailed information on the analytical process and quality control has been described elsewhere [22]. Blood samples were diluted using the modified alkali method to achieve a mixture density of 0.999 g/mL; therefore, “ng/g” units are approximately equal to “µg/L” units.

### Oral examinations

One trained dentist assessed the status of all the erupted primary teeth using a graduated mouth mirror (HD MIRRORS; Hu-Friedy, Chicago, IL, USA) under sufficient illumination. The dentist and participants sat facing each other in a knee-to-knee position during the checkup. In accordance with the protocol proposed by the Japan Association of School Dentists [23], the status of each primary tooth was categorized as sound, decayed (d), filled (f), or missing. The dft was calculated as the sum of the number of decayed and filled primary teeth. The dft numerically expresses the dental caries prevalence in the primary dentition, with larger values indicating higher prevalence. Dental plaque accumulation (three categories: score 0, little or no dental plaque on the anterior tooth surfaces; score 1, dental plaque covering ≤1/3 of the anterior tooth surfaces; and score 2, dental plaque covering >1/3 of the anterior tooth surfaces) and gingival status (three categories: score 0, no gingival inflammation; score 1, mild gingival inflammation; and score 2, moderate-to-severe inflammation) were assessed in the upper and lower anterior teeth. Because some categories included very few participants (1–3%), dental plaque accumulation was dichotomized as presence or absence of plaque, and gingival status as presence or absence of at least mild inflammation, for use in the analyses.

Data on professional topical fluoride application (yes or no), tooth brushing frequency (tooth brushing after every meal, yes or no), and parent-led tooth brushing (yes or no) were obtained through a questionnaire administered to the parents.

Data on participants’ age, sex, height, weight, and mother’s education level were obtained from health checkup records (JECS dataset: jecs-ta-20190930, jecs-qa-20210401). Percentage of overweight (POW), which is more commonly used in Japan as an indicator for childhood obesity evaluation than the body mass index-for-age percentile, was calculated as follows: POW (%) = 100 × (measured weight − standard weight)/standard weight [24, 25]. The standard weight is the age- and sex-specific weight for height, and the coefficients and formulas for the same have been described in detail elsewhere [25]. Participants were dichotomized as those with a POW ≥20% or <20% [24, 25]. The mother’s educational level was dichotomized as above or below high school level. Additionally, participants’ daily sucrose intake and household income at 4 and 6 years of age, respectively, were obtained from the JECS dataset. Participants’ daily sucrose intake at 4 years of age was estimated using the Brief Diet History Questionnaire (BDHQ). Although the BDHQ-estimated values are not as highly valid as absolute intakes, their validity for relative comparisons at the population level has been supported by a previous study [26]. Therefore, participants were categorized into tertiles according to the estimated values. Household income was categorized as follows: [1, <2.0 million yen; 2, 2.0–3.99 million yen; 3, 4.0–5.99 million yen; 4, 6.0–7.99 million yen; 5, 8.0–9.99 million yen; 6, 10.0–11.99 million yen; 7, 12.0–14.99 million yen; 8, 15.0–19.99 million yen; 9, ≥20.0 million yen]. Based on a previous study [27], we reclassified household income into two categories (<4.0 million yen and ≥4.0 million yen) for the analyses.

### Statistical analysis

First, the characteristics of the study population were described. Next, univariate and multivariate Poisson regression analyses with robust standard errors were conducted to examine the association between maternal blood lead levels during pregnancy and prevalence of dental caries in the primary dentition, expressed as dft, among the study participants. Natural log-transformed lead levels were used to correct for the skewed data distribution. The logarithm of the number of primary teeth was included as an offset variable in the regression analysis. Dental plaque accumulation, gingival status, age, sex, history of professional topical fluoride application, toothbrushing frequency, parent-led toothbrushing, POW, daily sucrose intake at 4 years of age, household income at 6 years of age, and mother’s educational level were selected as covariates. For sensitivity analysis, negative binomial regression was performed to assess the association between maternal blood lead levels during pregnancy and caries prevalence in the primary dentition. Statistical significance was set at p < 0.05. All statistical analyses were performed using Stata (version 18.0; Stata Corp LP, College Station, TX, USA).

## Results

### Participant characteristics

This study included 139 children, of whom 54.7% were girls, and 89.2% were 7 years old. The median maternal blood lead level was 6.1 ng/g (25–75 percentile, 5.0–7.3). The median dft was 0 (25–75 percentile, 0–4). Most participants did not exhibit plaque accumulation or gingival inflammation (Table 1).

**Table 1:** Participant characteristics (n = 139)

Maternal blood lead level (ng/g)	6.1 (5.0–7.3)
Oral examination	
dft	0 (0–4)
Number of primary teeth	14 (12–14)
Presence of dental plaque	14 (10.1%)
Presence of gingival inflammation	5 (3.6%)
Other characteristics	
Age	
7 years	124 (89.2%)
8 years	15 (10.8%)
Sex	
Girl	76 (54.7%)
Boy	63 (45.3%)
History of professional topical fluoride application	117 (84.2%)
Brushing teeth after every meal	59 (42.4%)
Parent-led toothbrushing	122 (87.8%)
Obesity (POW ≥ 20%)	6 (4.3%)
Daily sucrose intake at 4 years of age (g)	6.5 (5.1)
Household income at 6 years of age	
<4 million JPY	25 (18.0%)
Mother’s educational level	
High school level or below	35 (25.2%)

JPY, Japanese Yen; POW, percentage of overweight

Continuous variables are presented as medians with interquartile ranges (25th–75th percentiles), and categorical variables are presented as counts and percentages.

### Association between maternal blood lead levels during pregnancy and prevalence of dental caries in the primary dentition

Table 2 shows the results of the Poisson regression analysis for the association between maternal blood lead levels during pregnancy and dental caries in the primary dentition (numerically expressed as dft). In the crude model (univariate model), maternal blood lead levels were significantly associated with increased dft (prevalence ratio [PR], 1.2; 95% confidence interval [CI], 1.1–1.3; per one standard deviation increase in natural log-transformed maternal blood lead levels). This association remained statistically significant after adjusting for covariates, including age, sex, and oral health status and behavior. Adjusted PR (95% CI) was 1.6 (1.3–1.8). Additionally, poor gingival-health status, older age, male sex, being overweight (indicated by POW), higher sucrose intake, and lower mother’s education level were associated with a higher PR for dft. In contrast, history of professional application of topical fluoride and parent-led tooth brushing were associated with a lower PR for dft.

**Table 2:** Maternal blood lead levels during pregnancy and prevalence of caries in the primary dentition (n = 139)

Statistical approach = Poisson regression analysis with robust standard errors*						

**Outcome = dft**						

	**Univariable model**			**Multivariable model**		
	
	**PR**	**95% CI**	**p-value**	**PR**	**95% CI**	**p-value**
	
**Exposure variables**						
Maternal blood lead level (natural log-transformed) (per one SD increase)	1.2	1.1 to 1.3	<0.01	1.6	1.3 to 1.8	<0.01
Presence of dental plaque				1.4	0.8 to 2.4	0.26
Presence of gingival inflammation				116.7	64.6 to 210.8	<0.01
Age						
7 years				(reference)		
8 years				6.2	3.8 to 10.2	<0.01
Sex						
Girl				(reference)		
Boy				2.8	1.9 to 4.3	<0.01
History of professional topical fluoride application				0.5	0.3 to 0.7	<0.01
Brushing teeth after every meal				0.7	0.4 to 1.1	0.11
Parent-led toothbrushing				0.2	0.1 to 0.3	<0.01
Obesity (POW ≥ 20%)				11.4	5.8 to 22.4	<0.01
Daily sucrose intake at 4 years of age						
lower tertile				(reference)		
middle tertile				1.7	1.2 to 2.5	0.01
upper tertile				1.7	1.1 to 2.9	0.03
Household income at 6 years of age < 4 million JPY				1.0	0.6 to 1.5	0.87
Mother’s educational level: High school level or below				19.0	13.1 to 27.7	<0.01

*The logarithm of the number of primary teeth was included as an offset variable in the regression.

CI, confidence interval; POW, percentage of overweight; PR, prevalence ratio; SD, standard deviation.

The results of the sensitivity analysis using negative binomial regression models were consistent with those of the primary analysis. The crude and adjusted PRs (95% CIs) were 1.6 (1.0–2.4) and 1.8 (1.1–3.0), respectively (Table S1).

## Discussion

This cross-sectional study found that high maternal blood lead levels were associated with increased dft in children aged 7–8 years, suggesting that maternal exposure to lead during mid- to late-term pregnancy may affect the caries susceptibility of children after birth. To the best of our knowledge, this is the first study to show the relationship between maternal exposure to lead during pregnancy and the prevalence of dental caries in children after birth.

Previous studies have reported that lead can freely pass through the placenta, leading to its accumulation in the bones with a half-life of several decades, and that lead accumulated in the bones can migrate into circulating fluids and increase the blood lead levels [28]. Importantly, the primary cause of childhood exposure to lead is maternal-to-fetal transfer [28]. *In vivo* studies have shown that lead may be involved in the development of dental caries via several mechanisms. First, exposure to lead reduces salivary-gland function, including that of the parotid and submandibular glands, resulting in poor salivary flow [29]. This may result in demineralization owing to insufficient salivary buffering and other factors, such as reduced remineralization and increased acidity [29, 30]. Second, tooth formation consists of a two-step process: ameloblasts and odontoblasts secrete organic matrices, and calcification occurs as water and protein are removed from these matrices. Lead hampers this process by inhibiting enamel proteases, leading to delayed calcification and reduced enamel microhardness. Specifically, lead inhibits enamel formation by competitively antagonizing calcium and manganese and significantly reduces calcium and manganese content in hard tissues [17, 30]. In addition, lead inhibits local calcium metabolism, which potentially adversely affects the development and function of the odontoblasts [16, 31]. Based on these findings, the association between high maternal blood lead levels and increased prevalence of dental caries in the primary dentition observed in this study appears to be biologically plausible. The timing of maternal blood collection (i.e., the mid-to late-gestation period) was early in the period between the start (approximately 19 weeks of gestation) [32] and completion of calcification (i.e., 11 months postpartum), and lead migration from the mother affected susceptibility to dental caries in the primary dentition.

This study had several limitations. First, although our analyses adjusted for various potential confounding variables, residual confounding caused by unmeasured factors such as maternal nutrition or other unexpected variables cannot be ruled out. Second, in our study, the median gestational week at blood collection was 25.3 weeks (interquartile range, 24.6–26.6 weeks), according to the JECS protocol [33], whereas primary tooth germ development begins much earlier in the first trimester [34]. Thus, our measurements may not fully capture exposure during the earliest and most critical window of odontogenesis. Nevertheless, because lead has a relatively long half-life in bone and is continuously mobilized from maternal bone stores throughout pregnancy [35], mid-gestation blood levels are generally regarded as a reasonable proxy for overall prenatal exposure [33]. Third, the extent to which lead from maternal blood was transferred to the fetus was not evaluated in this study. Fourth, the study population was limited to 139 children residing in Kitakyushu City, Fukuoka Prefecture, which restricted both the sample size and the geographical representativeness. Although the sample size was relatively small compared with the nationwide JECS cohort and with previous studies such as those by Gemmel et al. (n = 543) [18] and Yepes et al. (n = 490) [36], we were able to detect statistically significant associations, suggesting that the observed effect size was sufficiently large to be detected despite limited statistical power. Nevertheless, the precision of our estimates should be interpreted with caution. Moreover, because our participants were recruited from a single urban area in Japan, the generalizability of our findings to other populations, including rural regions or non-Japanese populations, remains unclear. Environmental lead exposure, dietary patterns, and oral health practices may vary across geographical areas and cultures, and these differences could influence the magnitude or direction of the association. Importantly, unlike most prior studies that examined children’s blood lead levels, our study uniquely focused on maternal blood lead levels during pregnancy, a critical period for tooth germ formation. Thus, despite its smaller scale, our study provides novel evidence that complements previous epidemiological findings. Finally, because this was a cross-sectional study, we could not establish causality. However, some reports indicate that childhood blood lead levels are associated with increased dental caries incidence in young adults [30]. Therefore, further longitudinal follow-up studies are required to verify and expand upon our findings.

## Conclusions

In this study using data from the JECS, high maternal blood lead levels were associated with a higher prevalence of dental caries in the primary dentition. Thus, children of mothers with higher lead levels in the mid-to late-gestation period may be at risk for dental caries; therefore, extra attention should be paid to their oral health. The views expressed in this paper are the authors’ own and not those of the Ministry of the Environment.

## Abbreviations

CI: confidence interval
dft: decayed and filled teeth
PR: prevalence ratio
POW: percentage of overweight
JECS: Japan Environment and Children Study

## Supplementary information

**Additional file 1:** **Table S1.** Results of the negative binomial regression analysis for the association between maternal blood lead levels during pregnancy and prevalence of caries in the primary dentition (n = 139).

## Declarations

### Availability of data and material

The data are unsuitable for public deposition because of ethical restrictions and Japan’s legal framework. The Act on the Protection of Personal Information (Act No. 57 of May 30, 2003, amended on September 9, 2015) prohibits the public deposition of data containing personal information. The Ethical Guidelines for Medical and Health Research Involving Human Subjects enforced by the Japan Ministry of Education, Culture, Sports, Science, and Technology and the Ministry of Health, Labour, and Welfare also restrict open sharing of epidemiological data. All inquiries regarding access to the JECS Main Study data should be sent to jecs-en@nies.go.jp. The person responsible for handling inquiries sent to this e-mail address is Dr. Shoji F. Nakayama, JECS Program Office, National Institute for Environmental Studies. Oral examination data were specifically collected as part of this adjunct study to the JECS and are available from the authors upon reasonable request.
