# Supplementary material for: Association between maternal blood lead levels and prevalence of dental caries in the primary dentition of children
Source: Environ Health Prev Med. 2025 Nov 22;30:92. doi: 10.1265/ehpm.25-00188 (PMC12665915; doi:10.1265/ehpm.25-00188)
Supplement: Supplementary file 1 — Additional file 1: Table S1. Results of the negative binomial regression analysis for the association between maternal blood lead levels during pregnancy and prevalence of caries in the primary dentition (n = 139). [file ehpm-30-092-s001.docx]

**Supplemental material**

**Title**

Association between maternal blood lead levels and prevalence of dental caries in the primary dentition of children

**Authors**

Yoshie Nagai-Yoshioka^1^, Ryota Yamasaki^1^, Reiko Suga^2^, Mayumi Tsuji^3^, Reiji Fukano^4^, Kiyoshi Yoshino^5^, Seiichi Morokuma^6^, Wataru Ariyoshi^1^, Masanori Iwasaki^7*^

^1^ Department of Health Promotion, Division of Infections and Molecular Biology, Kyushu Dental University, Kitakyushu City, Japan

^2^ Regional Center for Japan Environment and Children's Study (JECS), University of Occupational and Environmental Health, Kitakyushu City, Japan

^3^ Department of Environmental Health, School of Medicine, University of Occupational and Environmental Health, Kitakyushu City, Japan

^4^ Department of Pediatrics, School of Medicine, University of Occupational and Environmental Health, Kitakyushu City, Japan

^5^ Department of Obstetrics and Gynecology, School of Medicine, University of Occupational and Environmental Health, Kitakyushu City, Japan

^6^ Department of Health Sciences, Graduate School of Medical Sciences, Kyushu University, Fukuoka City, Japan.

^7^ Department of Preventive Dentistry, Faculty of Dental Medicine and Graduate School of Dental Medicine, Hokkaido University, Sapporo City, Japan

**CONTENTS**

**Supplementary table**

**Table S1.** Results of the negative binomial regression analysis for the association between maternal blood lead levels during pregnancy and prevalence of caries in the primary dentition (n=139)

| Statistical approach=Negative binomial regression analysis* | | | | | | | | | | | |
| --- | --- | --- | --- | --- | --- | --- | --- | --- | --- | --- | --- |
| Outcome=dft | | | | | | | | | | | |
|  | Univariable model | | | | |  | Multivariable model^†^ | | | | |
|  | PR | 95% CI | | | p-value |  | PR | 95% CI | | | p-value |
| Exposure variable |  |  |  |  |  |  |  |  |  |  |  |
| Maternal blood lead level  (natural log-transformed)  (per one SD increase) | 1.6 | 1.0 | to | 2.4 | <0.01 |  | 1.8 | 1.1 | to | 3.0 | 0.02 |

*The logarithm of the number of primary teeth was included as an offset variable in the regression.

^†^Adjusted for dental plaque accumulation, gingival status, age, sex, professional topical fluoride application, toothbrushing frequency, parent-led toothbrushing, percentage of overweight, daily sucrose intake at 4 years of age, household income at the age of 6 years, and maternal educational level

CI, confidence interval; PR, prevalence ratio; SD, standard deviation.
